# Factors Controlling the Redox Potential of ZnCe_6_ in an Engineered Bacterioferritin Photochemical ‘Reaction Centre’

**DOI:** 10.1371/journal.pone.0068421

**Published:** 2013-07-30

**Authors:** Abdullah Mahboob, Serguei Vassiliev, Prashanth K. Poddutoori, Art van der Est, Doug Bruce

**Affiliations:** 1 Department of Biological Sciences, Brock University, St. Catharines, Ontario, Canada; 2 Department of Chemistry, Brock University, St. Catharines, Ontario, Canada; Center for Genomic Regulation, Spain

## Abstract

Photosystem II (PSII) of photosynthesis has the unique ability to photochemically oxidize water. Recently an engineered bacterioferritin photochemical ‘reaction centre’ (BFR-RC) using a zinc chlorin pigment (ZnCe_6_) in place of its native heme has been shown to photo-oxidize bound manganese ions through a tyrosine residue, thus mimicking two of the key reactions on the electron donor side of PSII. To understand the mechanism of tyrosine oxidation in BFR-RCs, and explore the possibility of water oxidation in such a system we have built an atomic-level model of the BFR-RC using ONIOM methodology. We studied the influence of axial ligands and carboxyl groups on the oxidation potential of ZnCe_6_ using DFT theory, and finally calculated the shift of the redox potential of ZnCe_6_ in the BFR-RC protein using the multi-conformational molecular mechanics–Poisson-Boltzmann approach. According to our calculations, the redox potential for the first oxidation of ZnCe_6_ in the BRF-RC protein is only 0.57 V, too low to oxidize tyrosine. We suggest that the observed tyrosine oxidation in BRF-RC could be driven by the ZnCe_6_ di-cation. In order to increase the efficiency of tyrosine oxidation, and ultimately oxidize water, the first potential of ZnCe_6_ would have to attain a value in excess of 0.8 V. We discuss the possibilities for modifying the BFR-RC to achieve this goal.

## Introduction

The water splitting reaction of photosynthesis has been the most influential biologically catalyzed reaction on earth. Acquiring the ability to use water as a source of electrons about 2.5 billion years ago allowed oxygenic photosynthesis to power a massive increase in the diversity and numbers of aerobic life forms. Photosystem II (PSII) is the pigment-protein complex embedded in the thylakoid membranes of plant chloroplasts and cyanobacteria that catalyses the light induced oxidation of water and reduction of plastoquinone in oxygenic photosynthesis [1], [2]. This catalytic function is performed by light driven electron transfer (ET) reactions through redox cofactors of the PSII reaction center. Oxidation of water by PSII requires several essential cofactors: a photoactive strongly oxidizing pigment (P680), a redox-active tyrosine (Y_Z_) and the oxygen-evolving complex (OEC) containing four high-valence manganese ions bound by μ-oxo bridges and a calcium ion [2], [3]. Electronic excitation of P680 and subsequent electron transfer to the primary pheophytin electron acceptor forms the strongly oxidizing P680^•+^ cation radical (E_m_∼1.12 V) which then oxidizes Y_Z_. Subsequently, Y_Z_ oxidizes the OEC where water oxidation occurs after the accumulation of four oxidation equivalents in the Mn_4_CaO_5_ cluster according to the S-cycle proposed by Joliot and Kok [4], [5]. The coupling of water oxidation to photochemistry in PSII was a crucial milestone in the evolution of life allowing for the essentially unlimited conversion of sunlight energy to chemical potential energy which now powers most life on earth. The successful mimicking of this reaction in an artificial system could form the basis of a clean alternative energy source. Recently, bacterioferritin (cytochrome b1, BFR) was used as a protein scaffold for constructing a linear electron pathway that mimics some of the electron transfer components within PSII [6].

**Figure 1:**
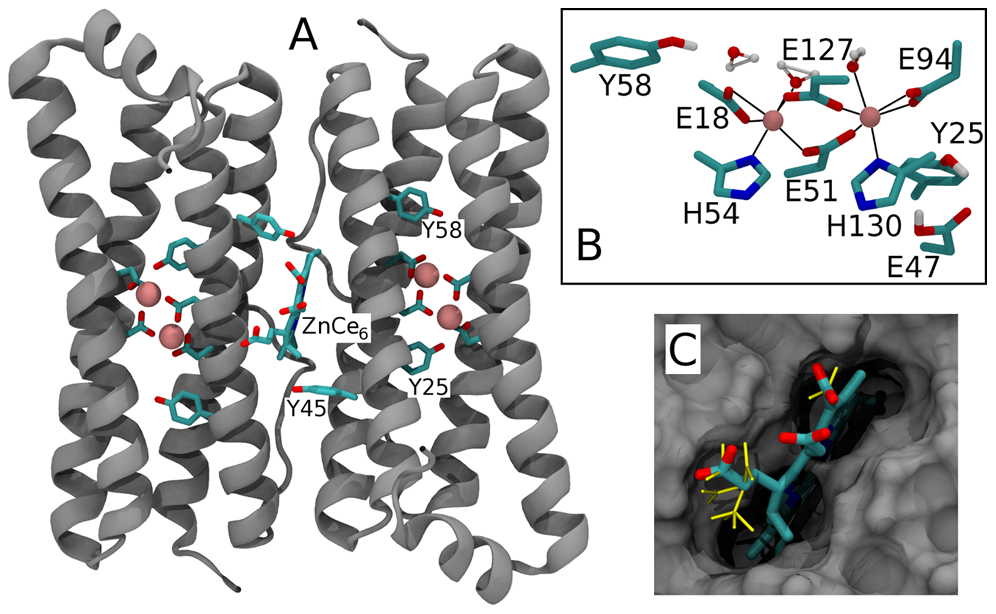
Atomic level model of the *E. coli* bacterioferritin. A - homodimer showing two identical subunits each hosting two manganese ions and ZnCe_6_ bound at the interface of the subunits. B – manganese binding site. C - ZnCe_6_ in its binding site. The ensemble of carboxylic acid conformations used to compute pKas is shown in yellow. Model is based on the X-ray diffraction structure PDB ID: 3E1M.

Ferritins are the principal iron storage proteins in most living organisms [7]–[9]. The protein BFR is a robust iron storage bacterial protein that forms a homodimer, with each subunit (∼18.5 kDa) being composed of an antiparallel, four-helical bundle [10]–[12] (**Fig. 1**). The homodimers self-assemble into a dodecamer that forms a spherically shaped protein shell surrounding an internal cavity encapsulating an iron core composed of ferric hydroxyphosphate micelles [10]. Each BFR subunit binds 2 iron ions (Fe^2+^) and contains seven tyrosine residues [10], [12]. The BFR dimer binds one b-type heme at the interface between subunits [11]. In each subunit three tyrosines are located close to both metal ions and the heme (**Fig. 1**). Thus, a protein scaffold of BFR is suitable for constructing a photoactive reaction center mimicking the electron transfer reactions of PSII. A prototype of such a ‘reaction center’ (BFR-RC) has been created by replacing the heme with a photoactive zinc-chlorin-e_6_ (ZnCe_6_) pigment and the iron ions with Mn(II) ions [6]. It has been found that the bound ZnCe_6_ species are capable of initiating electron transfer upon illumination, oxidizing a tyrosine residue and the bound manganese Mn(II) ions [6].

The mechanism of the light activated electron transfer observed in BFR-RC, however, remains poorly understood. It is not clear whether photooxidation of tyrosine and the bound manganese are sequential or independent events. One of the central questions key to understanding ET pathways, overall efficiency and limitations of BFR-RC is the unknown value of the oxidation potential of the ZnCe_6_ cation radical in BFR-RC. The E_m_ of ZnCe_6_ provides the driving force for oxidation of cofactors and its value will determine which mechanisms of tyrosine oxidation are possible and whether this BFR-RC may eventually be able to oxidize water. To our knowledge the oxidation potential of ZnCe_6_ has not been measured experimentally in either solvent or protein. The present article investigates the oxidation and reduction potentials of ZnCe_6_ in solution and in BFR-RC with a combination of experimental and computational methods. Our focus is on understanding factors influencing the redox potential of ZnCe_6_ within BFR-RC with an aim to identify possible means of controlling it.

## Materials and Methods

### Synthesis of ZnCe_6_

ZnCe_6_ was prepared by metallating chlorin e_6_ with Zn(OAc)_2_ by standard methods [13]. Typically, 40 mg (0.067 mmol) of chlorin e_6_ and 37 mg (0.20 mmol) of Zn(OAc)_2_ were stirred at room temperature in a CHCl_3_/CH_3_OH (8/4 ml) solvent mixture. The reaction was monitored by absorption spectroscopy. A red shift of the Q_Y_ absorption band from 661 to 640 nm accompanied incorporation of zinc. We also confirmed formation of ZnCe_6_ by observing the disappearance of the free base pigment protons by NMR spectroscopy. After completion of metallation the solvents were evaporated and the residue was washed with water and cooled methanol to get spectroscopic grade pure compound. The yield of reaction was 39 mg (89%).

### Electrochemical measurements

Differential pulse voltammetric measurements were performed in pyridine and DMSO containing 0.1 M tetrabutylammoniumhexafluorophosphate (TBAPF_6_) on a BAS Epsilon electrochemical analyzer (working: Pt, auxiliary electrodes: Pt wire; reference electrode: Ag). The pulse width, period and amplitude used were 50 ms, 200 ms and 50 mV, respectively. Sample concentration was 1 mM and ∼100 fold excess of N-methylimidazole was added to prevent aggregation between ZnCe_6_ molecules. Resultant solutions were purged with nitrogen gas for 10 min prior to the scan. The ferrocene/ferrocenium couple was used to calibrate the redox potential values. All experiments were performed at 296 K. The use of water as solvent for electrochemical measurements was avoided to eliminate reactions of cations and di-cations with water and any changes in E_m_ which could arise from ionization of ZnCe_6_ carboxylic acid groups.

Spectroelectrochemical measurements were performed at a platinum mesh electrode in a thin layer spectroelectrochemical cell (0.5 mm). Potentials were applied and monitored using the same potentiostat as for the differential pulse voltammetric measurements. Absorption spectra were recorded with an Ocean Optics USB650 Red Tide spectrometer.

### Calculation of the shifts to the redox potential of ZnCe_6_ due to coordination to axial ligands, ring substituent groups, and dielectric constant

To calculate the shift to the redox potential due to axial ligation for the ZnCe_6_^+^/ZnCe_6_ couple, we performed computations using density functional theory (DFT). The B3-LYP functional with the LanL2TZ+ basis set for the Zn atom and 6-311G+** for C, H, N and O were used. Gas-phase zero-point energies, thermal corrections, and entropic corrections were calculated using standard formulas for the statistical thermodynamics of an ideal gas using optimized geometries and scaled by 0.9613 B3-LYP/6-31G*/LanL2TZ+ frequencies [14]. Solvation energies of the studied species in the various solvents were calculated using the solvation model SM8 [15] at the B3-LYP/6-31G* level of theory. In all solvation energy calculations the LanL2TZ+ basis set was used for Zn.

### Modeling BFR-RC structure

The X-ray bacterioferritin structure, (PDB ID: 3E1M), from *E. coli* with a resolution of 2.7 Å was used as a starting point for calculations. The structure was modified to match an engineered BFR-RC described in [6], [16]. Two surface exposed histidines (H46, H112) were mutated to arginines, the heme was replaced with photoactive ZnCe_6_ and iron ions were replaced with Mn(II) ions.

In BFR protein the heme binds in a symmetrical hydrophobic pocket located on a twofold axis between symmetry related subunits. As the structure of ZnCe_6_ is similar to the structure of *b*-type heme (ZnCe_6_ has formyl, acetyl and propyl groups at positions 13, 15 and 17 and saturated ring IV while heme has propyl groups at 13 and 17 and all rings unsaturated) we placed ZnCe_6_ in the same position and orientation as heme in the BFR. After initial placement of ZnCe_6_ in its binding pocket, conformations of the three carboxylic groups of ZnCe_6_ were optimized using a Monte-Carlo search as implemented in MCCE. The highest occupancy conformers were then used as a starting structure for a complete energy minimization of the whole BFR-RC with constrained backbone atoms which was then followed by a short 10-ps unconstrained molecular dynamics run. AMBER version 10 [17] was used for molecular mechanics computations. The “pairwise” generalized Born implicit solvent model [18] was used in these simulations. Initially we used standard ionization states for all aminoacids, and all 3 carboxylic groups of ZnCe_6_ were protonated. The final RMSD of the protein backbone between the original PDB ID: 3E1M structure and BRF-RC was 0.15 Å. Next we performed ONIOM QM/MM geometry optimization of the whole protein with ZnCe_6_ and its methionine ligands treated quantum mechanically at the B3LYP/6-31g* level using Gaussian 09 [19]. In QM/MM optimized structures both methionine ligands were almost equidistant from Zn. The equilibrium distances between Zn and S were found to be 2.80/2.85 Å. The QM/MM optimized structures of neutral and radical ZnCe_6_ with its 2 methionine ligands were used to refine atom-centered point charges obtained from the initial model. The optimized structure of the BFR-RC with bound ZnCe_6_ is available in PDB format in supporting **Model S1**.

### Derivation of the atomic partial charges for ZnCe_6_

The atomic partial charges for ZnCe_6_ were obtained using the two stage RESP formalism [20], with a weighting factor of 0.0005/0.001 from a wavefunction computed at the HF/6-31G* level for H, N, C, O, and HF/LanL2TZ+ for Zn. Schematic diagram of ZnCe_6_ is shown in **Fig. S1**, atomic point charges for ZnCe_6_ are available in **Table S1**. QM calculations were done with the Gaussian09 package [19].

### Computation of protonation pattern and shift of the redox potential of ZnCe_6_ in BFR-RC

MCCE2.4 [21] was used to predict the ionization state of each protein residue and cofactor as a function of E_h_ and pH. The algorithm performs Monte-Carlo sampling of multiple aminoacid side chain geometric and ionization conformations, where conformer energies include electrostatic and van der Waals terms. Before sampling, rotamers of aminoacid side chains were generated using 60 degree increments for each rotatable bond, while conformers of carboxylic acid groups of ZnCe_6_ were generated in 14 degrees increments. Pairs of conformers with clashes not exceeding 5 kcal/mol had their positions optimized. Finally, a genetic algorithm was used to optimize side chain conformers [22]. Electrostatic conformer-conformer pairwise interactions and reaction field energy for each conformer were computed by solving the linearized Poisson-Boltzmann equation using the Delphi program [23]. The dielectric constant was set to 4 inside the protein and 80 in the solvent. PARSE [24] radii and charges were used in Poisson-Boltzmann calculations for all elements except Zn. For Zn, a radius of 1.47 Å was used [25]. To obtain the E_m_ of ZnCe_6_ in BRF-RC the shift of the redox potential in the protein was added to the E_m_ of the reference model system.

In calculations of the shift of E_m_ in a protein environment several factors were considered: (i) the desolvation energy difference arising from moving the cofactor from water into protein , where the protein volume is simply considered as dielectric medium ΔΔ*G_desolv_*, (ii) the electrostatic and VDW non-electrostatic interaction of the cofactor with the protein backbone Δ*G_bkbn_*, and (iii) the mean field pairwise interaction between the cofactor and side chains of residues in the protein in the distribution derived by Monte Carlo sampling 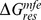. The difference in each energy term is between the oxidized and the reduced cofactor. Details of the calculations of these factors are described in [26].

In continuum electrostatics calculations ZnCe_6_ and side chains of its methionine axial ligands were represented as a single residue with an oxidized or reduced conformer. The backbone atoms of the axial ligands remained as part of the protein backbone. QM treatment of the complex of ZnCe_6_’ (ZnCe_6_ without carboxylic groups) with 2 methionine axial ligands yielded E_m(sol)_ of 0.58 mV (see Results section 3.3) which was used as the reference for MCCE calculations. The three carboxylic groups of ZnCe_6_ were treated as independently ionizable groups.

## Results

### Oxidation and reduction potentials of ZnCe_6_ in solution

**Figure 2:**
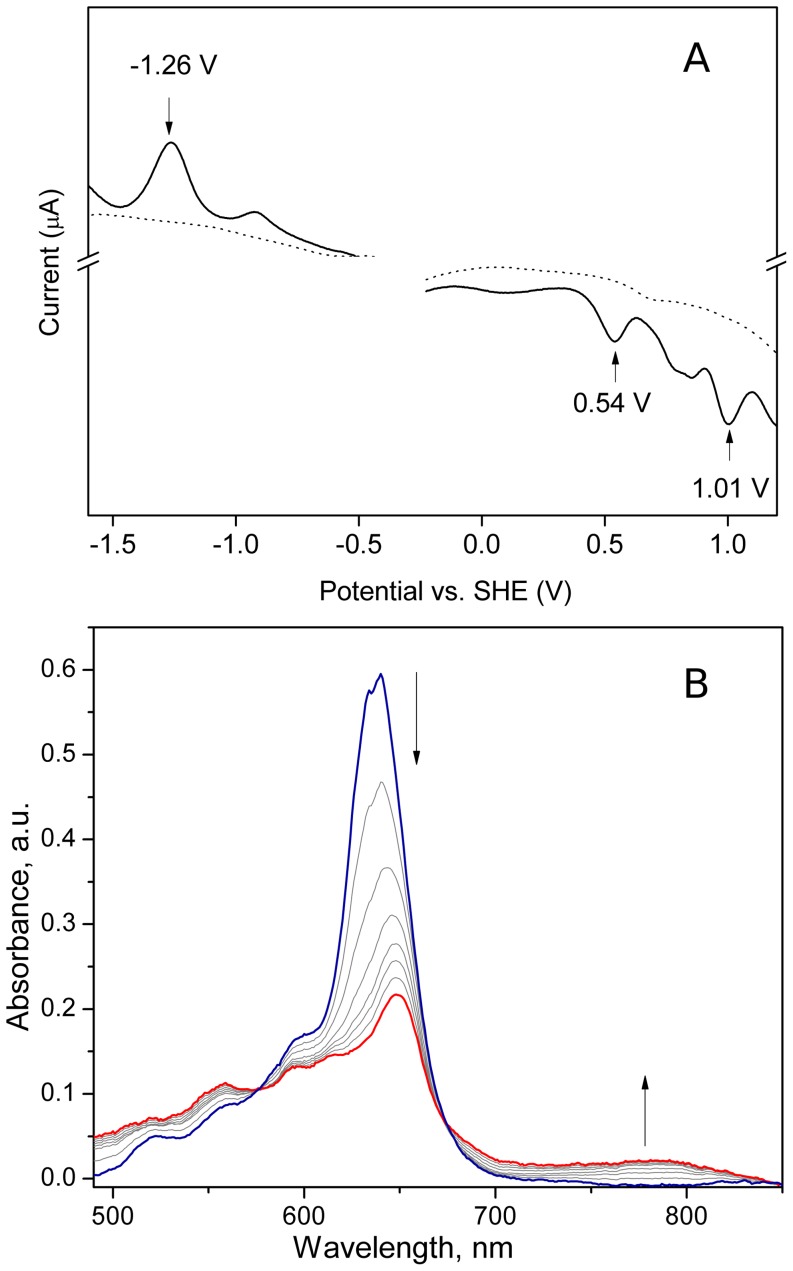
A: Differential pulse voltammogram of ZnCe_6_ in DMSO with 0.1 M TBAPF_6_. [ZnCe_6_] = 1 mM. Voltammogram of the solvent is shown by dotted line. B: Spectral changes during the oxidation of ZnCe_6_ at 0.54 V. See Results for details.

ZnCe_6_ has low solubility in most organic solvents. We were able to measure its redox potential in only two solvents (pyridine and DMSO). Although we observed oxidation waves in both solvents, only with N-methylimidazole were two clear waves visible. Voltammograms measured in DMSO without N-methylimidazole showed multiple redox waves (data not shown), suggesting that under these conditions several different aggregation states of ZnCe_6_ co-existed in the sample. Aggregation occured most likely due to the coordination of Zn by Lewis base atoms of the substituent carboxyl groups. These aggregates can be broken down by addition of stronger Lewis bases such as N-methylimidazole. Indeed, after addition of N-methylimidazole to the ZnCe_6_/DMSO solution, two distinctive redox couples appeared in the oxidative scan. The major oxidation peaks were centered at 0.54 and 1.01 V vs. standard hydrogen electrode (SHE) (**Fig. 2A**). In addition a smaller and broader feature was observed at 0.8 V. The spectral changes obtained during oxidation of ZnCe_6_ at 0.54 V are shown in **Fig. 2B**. The Q_Y_ band of the neutral compound at 640 nm decreased in intensity while a new absorption band grew at around 800 nm. These spectral features can clearly be assigned to a chlorin π cation radical. The difference between the first and the second oxidation of methylimidazole - coordinated zinc porphyrins has been reported to be in the range of 0.46–0.66 V [27]. In our voltammogram the peak centered at 1.01 V, matching this difference, was assigned to a two electron oxidation of ZnCe_6_. There was one redox couple in the reductive scan at −1.26 V tentatively assigned to the formation of the ZnCe_6_ anion radical (**Fig. 2**).

### Effect of axial ligands and carboxyl groups on the oxidation potential of ZnCe_6_ in solution

To calculate the E_m_ of ZnCe_6_ in BFR we needed a reference E_m_ corresponding to ZnCe_6_ ligated by methionine without carboxyl groups in water. To obtain this value from the measured E_m_ of ZnCe_6_ in DMSO we computed shifts to the E_m_ due to different solvents, axial ligands and carboxyl groups.

Axial ligands are known to induce changes in the electrochemistry of metalloporphyrins and metallochlorins [27], [28]. For example, it was found that upon imidazole ligation the first oxidation potential of zinc porphyrins shifts negatively by 150 mV in CH_2_Cl_2_ [27]. In contrast the second oxidation potential of complexed zinc porphyrins shifted positively by 50–270 mV when compared with the uncomplexed zinc porphyrins depending on the nature of the substituents [27].

Our DFT calculations showed that N-methylimidazole shifts the first oxidation potential of ZnCe_6_ in water negatively by 70 mV, while the coordination to 2 methionines had a smaller negative shift of only 20 mV (**Table 1**). This trend is similar to the results of previous computational work on chlorophyll *a* [28].

**Table 1:** Oxidation potentials of the ZnCe_6_ and ZnCe_6_ without carboxylic acids in continuum solvents with different values for the dielectric constant.

Axial ligand	ZnCe_6_ w/o carboxylic groups			ZnCe_6_		
	Gas (ε = 1)	DMSO (ε = 46.8)	Water (ε = 78.4)	Gas (ε = 1)	DMSO (ε = 46.8)	Water (ε = 78.4)
No	1.54	0.44	0.51	1.59	0.55	0.64
2 Met	1.32	0.35	0.48	1.36	0.47	0.62
Imidazole	1.15	0.36	0.44	1.20	0.48 (0.54^a^)	0.57

a - experimentally measured value.

A second factor affecting the oxidation potential is the nature of ring substituent groups [29], [30]. In general, electron withdrawing groups shift the oxidation potential up while electron rich groups shift it down. Protonated carboxyl groups, being electron withdrawing, are expected to up-shift the redox potential. This effect is pH dependent as ionization of carboxyl groups stabilizes the cation radical, and hence will down-shift the redox potential.

To estimate the shift of the redox potential of ZnCe_6_ in BFR-RC protein we treated the carboxyl substituent groups and Zn–chlorin as separate units, affecting each other via classical electrostatic and VDW interactions. This approach allowed us to sample efficiently multiple conformational and ionization states of carboxylic acids. A similar approach was previously successfully used for calculation of heme redox potentials in different proteins [31]. These calculations require knowledge of the reference redox potential of Zn–chlorin without carboxyl groups in water, while experimentally we measured ZnCe_6_ with three substituent carboxylic groups in DMSO. Therefore, to obtain the reference redox potential of Zn–chlorin we estimated differences in redox potential due to solvent and the presence of carboxyl groups computationally.

Our DFT calculations showed that the redox potential of both coordinated and non-coordinated ZnCe_6_ changes insignificantly between DMSO and water. This result is consistent with previous experimental and theoretical studies of chlorophyll *a* [32] in solvents with different dielectric constant. However, the attachment of protonated carboxyl groups to Zn-chlorin in different coordination states leads to an increase of the redox potential by 0.13–0.14 V in water.

To estimate the accuracy of our DFT calculations we determined the E_m_s of 10 different zinc chlorins and compared them to experimental values [30]. All computed values were systematically lower than the experimental values by 0.1 V. After correction for this systematic shift, the computed E_m_s were within 0.03 V of the experimental values (**Fig. S2**). Overall, the reference oxidation potential corresponding to the first oxidation of bis-methionine coordinated ZnCe_6_ in water without carboxylic groups was estimated to be 0.58 V vs. SHE.

### Oxidation potential of ZnCe_6_ in BFR-RC

In BFR-RC ZnCe_6_ is partially buried in the protein. Our Monte-Carlo simulations estimate that desolvation due to embedding into the protein's lower dielectric increases the E_m_ of ZnCe_6_ by 0.07 V. Another modest increase of the E_m_ arises from interaction with dipoles of the protein backbone (0.05 V). In contrast the side chains of aminoacids (including the ZnCe_6_ carboxylic groups) decrease the redox potential (**Table 2**).

**Table 2:** Effect of ionization state of three carboxylic groups of ZnCe_6_ on its E_m_.

	*E_m(sol)_*	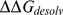	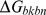	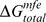	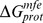	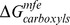	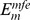
All 3 titrated	0.58	0.07	0.05	−0.13	−0.10	−0.04	0.57
All 3 neutral	0.58	0.07	0.05	−0.03	−0.12	0.08	0.67
All 3 ionized	0.58	0.07	0.05	−0.22	−0.08	−0.14	0.48

Computed at pH = 6.

The ionization state of the carboxyl groups is expected to affect the ZnCe_6_ redox potential. While neutral carboxyl groups increase E_m_ by 0.14 V in water (**Table 1**), ionized groups will stabilize the cationic form and shift the E_m_ down. In addition the redox state of ZnCe_6_ is bound to affect the pKas of its carboxyl groups. Oxidized ZnCe_6_ will tend to lower the pKas and increase ionization of the carboxyl groups. This would tend to further lower the ZnCe_6_ oxidation potential.

**Figure 3:**
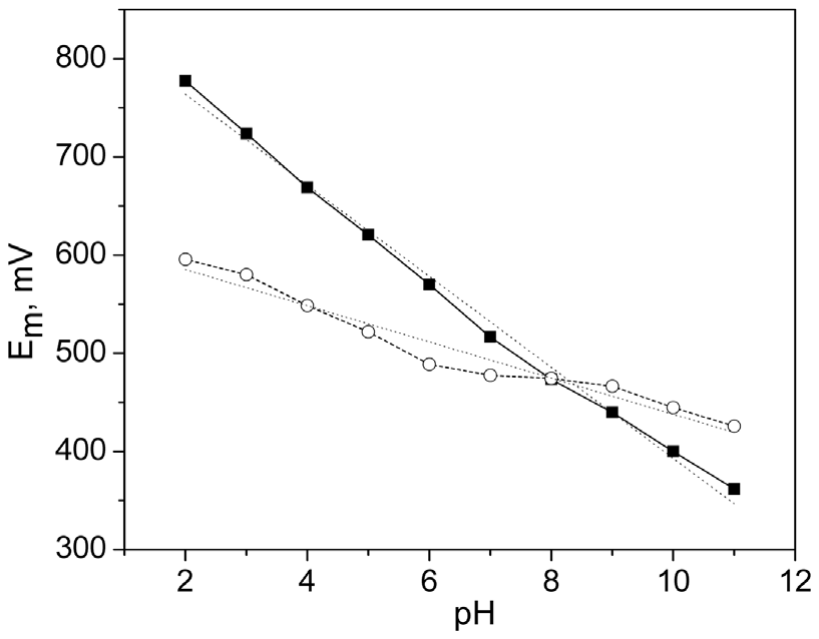
Calculated pH - dependence of the E_m_ of ZnCe_6_ in BFR-RC (solid line, slope −46.4, R = 0.994) and in water (dashed line, slope −18.4, R = 0.950).

The case when all carboxyl groups are protonated before oxidation and remain protonated after oxidation provides an upper bound for the ZnCe_6_ redox potential. With pKa values determined in the previous section, the charges of carboxylic groups with neutral ZnCe_6_ at pH 6 are: −0.25, −0.56, −0.88 for formyl, acyl and propyl respectively when all carboxylic groups were titrated together. The probability of fully protonated ZnCe_6_ (the product of occupancies of all 3 protonated groups) in this case is fairly low (4.4%). However, performing a titration of ZnCe_6_ while the propyl group is fully charged decreases the redox potential by only 0.02 V compared to an all-neutral titration. The charges of carboxyl groups with the cation radical of ZnCe_6_ at pH 6 increase to −0.93, −0.25, −0.93 for formyl, acyl and propyl respectively. The probability of a fully protonated cationic form of ZnCe_6_ is very low. If all carboxylic groups are protonated in both cationic and neutral forms, the ZnCe_6_ has an E_m_ of 0.67 V (**Table 2**). This result gives an idea of how high the E_m_ of ZnCe_6_ in BFR-RC could be if the carboxyl groups were to be replaced by neutral groups. In another extreme, when all carboxyl groups are ionized, ZnCe_6_ has the lowest E_m_ of 0.48 V (**Table 2**). Of course the actual value of E_m_ is somewhere between 0.48 and 0.67 mV. In agreement with upper and lower bound estimates we obtained an E_m_ of 0.57 V at pH 6 in calculations when the ionization of titratable groups was sampled simultaneously with the redox titration of ZnCe_6_. Our calculations also showed that in the pH interval from 4 to 8, the E_m_ decreases by 0.21 V (**Fig. 3**).

**Figure 4:**
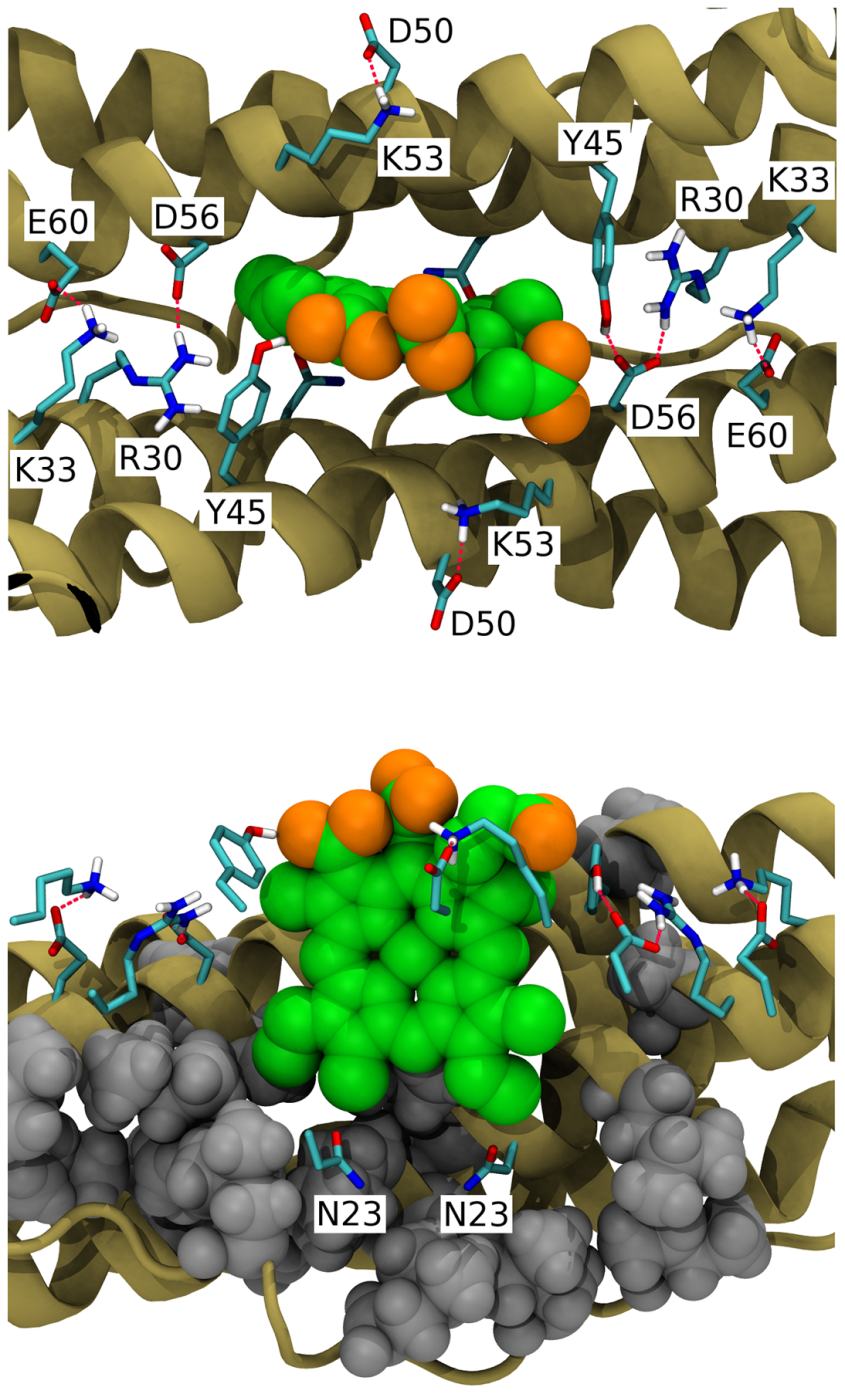
Aminoacids contributing the most to the E_m_ shift of ZnCe_6_. Grey spheres represent hydrophobic side chains.

Contributions to the E_m_ shift from individual aminoacids in the BFR-RC are listed in **Table 3**. Seven aminoacids, located close to ZnCe_6_, have the largest contributions to the E_m_ shift (**Fig. 4**). Four of these aminoacids form two pairs of salt bridges between two monomers, while one pair (Asp50-Lys53) forms a salt bridge within a single protein subunit. Although each of these charged aminoacids would have a large effect on E_m_ if considered separately, their participation in salt bridges neutralizes their effective charge greatly, thus decreasing their ability to shift the E_m_. Finally a large fraction of the decrease comes from Asn23. This aminoacid decreases E_m_ by −0.02 V in both monomers of the homodimer resulting in total shift of −0.04 V. The two manganese clusters contribute −0.02 mV each, and the longrange electrostatic contributions from the rest of the protein aminoacids decrease the E_m_ by −0.02 mV.

**Table 3:** Contributions from aminoacid side chains to the shift of ZnCe_6_ E_m_.

Aminoacid	E_m_ shift	Aminoacid	E_m_ shift	Total
**Arg30**	**0.08**	**Asp56**	**−0.08**	0.00
**Lys 33**	**0.01**	**Glu 60**	**−0.02**	−0.01
**Lys 53**	**0.03**	**Asp 50**	**−0.02**	0.01
**Asn 23**	−0.04			−0.04
**Others**				−0.05

Computed at pH = 6. Aminoacid pairs participating in salt bridges between chains A and B are shown in bold in the one row. The total E_m_ shift, computed as sum of both monomers is show.

### Oxidation potentials of tyrosines in BFR-RC

Three tyrosines in BRF-RC are located near ZnCe_6_ and the di-metal center. Tyr25, 45 and 58 are found at 3.8/10.6, 13.7/4.5 and 5.8/10.4 Å from the di-metal center/ZnCe_6_ respectively. The four other tyrosines are located at the periphery of the protein far from both cofactors. After performing Monte-Carlo sampling of protonated neutral and protonated cationic radical species to determine the redox potential of each tyrosine in BFR-RC we found that Tyr25 has the lowest oxidation potential.

**Figure 5:**
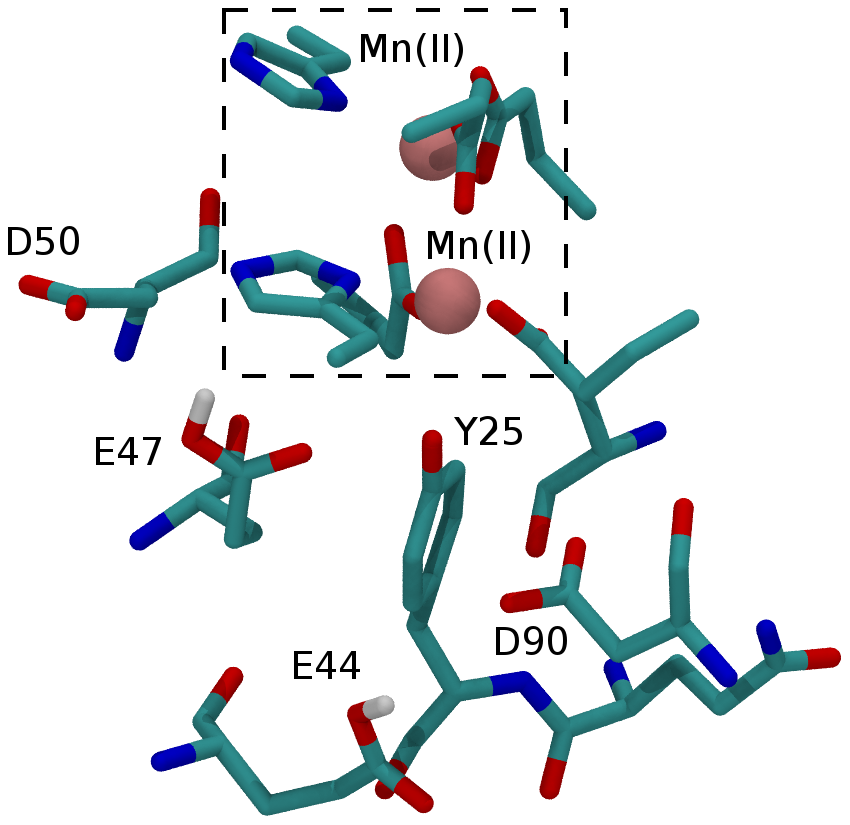
Details of the protein environment of Tyr25.

The decrease of Tyr25's oxidation potential arises from the high polarity of its immediate environment which stabilizes the Tyr25 cation radical. The protein environment of Tyr25 is shown in **Fig. 5**. The polarity of the Tyr25 environment is reflected by increase in its pKa of ∼10 units. Most of this shift is due to Asp90 and Glu47. The presence of these two negatively charged amino acids in the vicinity of Tyr25 destabilizes the deprotonated form of Tyr25, while the positively charged radical is favoured.

Our Monte-Carlo simulations indicated that at pH 8 the Tyr25 oxidation potential is decreased in BFR-RC by 160 mV relative to the tyrosine reference potential of 1380 mV in solution. The oxidation potentials of all other tyrosines in BFR-RC are increased by >250 mV. The largest contributions to the E_m_ shift of Tyr25 are from Asp90 (−300 mV), the di-metal center with its ligands (−108 mV), Glu47 (−50 mV), Glu44 (30 mV) and Asp50 (−40 mV). Glu44, Glu47 and Asp90 are located approximately equidistantly from Tyr25, but only Asp90 has a large effect on its oxidation potential. This difference is because only Asp90 is fully ionized. Occupancy of the charged species of Glu47 is 0.2, and Glu44 which has the lowest influence on the shift of Tyr25 E_m_ is neutral. The occupancy of the neutral species of both Glu47 and Glu44 is high because both of them are located in a hydrophobic environment (Leu40, Leu87, Leu134, Trp133). Despite the decreased oxidation potential of Tyr25 in BFR-RC (1220 mV) it is still too high for a cation of ZnCe_6_ to oxidize.

## Discussion

The E_m_ of ZnCe_6_ in DMSO is 0.54 V and our calculations predict that it remains not far from this value when ZnCe_6_ is embedded in BFR-RC. This E_m_ value is significantly lower than was anticipated [6], and well below that required to oxidize water. It also raises the question of how ZnCe_6_ oxidizes tyrosine in the BFR-RC, as in all known cases the redox potential of tyrosine in proteins is near 1 V [33]–[35]. However, light-induced tyrosine oxidation has been observed in BFR-RC [6]. How can tyrosine be oxidized by ZnCe_6_?

In PSII there are three possible mechanisms for the oxidation of tyrosine in the redox reaction between P680 and Mn-cluster (reviewed in [36]):
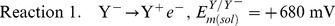


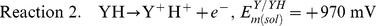


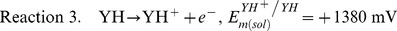
Where species of tyrosine are denoted as: YH, protonated neutral; 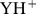, protonated cationic radical; 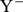, deprotonated anion; 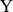, deprotonated neutral radical. These standard potentials were measured for N-acetyl-_L_-tyrosinamide in aqueous solutions [36], [37]. In aqueous solution, the pathway of tyrosine oxidation depends predominantly on pH. In a protein, these reactions can also be controlled by hydrogen bonds or electrostatic interactions of tyrosine with its local environment.

Our computations suggest that none of the above reactions could be driven by photooxidation of ZnCe_6_. Reaction 1 requires the redox active tyrosine to be ionized. Tyrosine ionization can only occur if positive charges near the tyrosine significantly lower its pKa. None of the tyrosines in BFR-RC satisfy this requirement.

Reaction 2 requires a somewhat higher oxidation potential as compared to reaction 1. In solution Tyr is oxidized by this mechanism at +970 mV. Reaction 2 is proton-coupled and requires a suitable proton acceptor e.g. a nearby His, able to bind the proton released by tyrosine. None of the tyrosines in BFR-RC have suitable His proton acceptors nearby. However, we cannot exclude that water hydroxyls in specific hydrogen bond networks may accept protons from tyrosine.

In solution reaction 3 occurs at +1380 mV, in PSII the calculated E_m_ for this reaction is raised to +1576 mV [35]. We estimated that Tyr25 has the lowest oxidation potential of all tyrosines in BFR-RC, its E_m_ is around 1220 mV. Our calculations indicate that the single oxidized ZnCe_6_ with oxidation potential of 570 mV at pH 6 would not be capable of tyrosine oxidation. Considering that a tyrosine cation radical EPR signal in BFR-RC has been observed after a prolonged exposure to saturating light or several saturating laser flashes [16], [38] we suggest that a photogenerated ZnCe_6_ di-cation [39] may be responsible for oxidizing tyrosine. The ZnCe_6_ di-cation, having a solution value of 1022 mV, would have a sufficiently high E_m_ to oxidize tyrosine in BFR-RC after taking protein effects into account.

It is clear from our results that the relatively low oxidation potential of ZnCe_6_ limits its use in the construction of a BFR-RC that would eventually be capable of oxidizing water. What factors could help increase the oxidation potential of ZnCe_6_? Oxidation potentials of reaction center chlorophylls in photosynthetic organisms span a wide range from only 500 mV for P700 in PSI [40] and P870 in purple bacteria [41] to 1,100–1,200 mV for P680 in PSII [42], [43]. Several major factors give rise to the differences in E_m_ [44]. Most of the difference in E_m_ between reaction centers of PSI and PSII originates from the protein atomic charges and charges of cofactors. Together they up-shift the E_m_ of P_D1_ in PSII by 325 mV, but, in contrast, shift the *E*_m_ of P_A_ in PSI down by −125 mV [44].

One of the major factors raising the E_m_ of P680 in PSII is the unique Mn_4_Ca cluster, bearing a large positive charge. This cluster alone shifts the E_m_ of P_D1_ in PSII up by 214 mV [44]. The BFR-RC has two di-manganese centers composed of Mn(II) ions. Each of the di-manganese centers shifts the E_m_ of ZnCe_6_ up by 85 mV. However, this effect in BFR-RC is compensated by the charged ligands to the manganese which down-shift the E_m_ by −90 mV. The combined effect of all aminoacid sidechains was found to shift the E_m_ of BFR-RC down by about −92 mV, not as much as the sidechains of PSI lacking the Mn_4_Ca cluster. It is likely, however, that this down-shift would become smaller and maybe even turn into an up-shift with photooxidation of Mn(II) ions and formation of μ-oxo bridges.

Another major factor contributing to the increase of the E_m_ in P680 in PSII is its position close to the luminal edge of two transmembrane α-helixes (helixes d_D1/D2_ providing axial His ligands to P_D1/D2_). The protein backbone dipole of helix d shifts the E_m_ of P_D1_ in PSII by about 95 mV. The corresponding helix in PSI (helix j) shifts the E_m_ of P_A_ down by 28 mV [44]. The organization of helices coordinating ZnCe_6_ in the BRF-RC is more similar to PSI than to PSII which is reflected by similar contributions from the backbones (40 mV).

The choice of ZnCe_6_ for the role of photoactive pigment is attractive because of its availability and solubility in water, however, our work demonstrated that this pigment is not capable of providing sufficient oxidative power for the water splitting reaction. One of the problems is the detrimental effect of ionized carboxyl groups on the redox potential. Our calculations indicate that a simple replacement of these groups with neutral groups would increase the E_m_ of ZnCe_6_ by 130 mV. Even higher potentials may be achieved by replacement of the acidic groups with basic groups.

Axial ligands to Zn are another factor known to affect redox potentials. For example, it has been shown that Met-Met coordination increases the potential of heme by about 200 mV compared to His-Met ligated heme [45]. In this aspect BFR-RC already has the best axial ligand, and replacing Met with His in BRF-RC would likely shift the potential down.

A large fraction of the E_m_ decrease observed in BFR-RC comes from interaction with the two Asp23 residues. Interestingly, this polar aminoacid is located in the hydrophobic region of the ZnCe_6_ binding pocket. In addition, this aminoacid is conserved among ferritins from several organisms (PDB ID: 2FKZ, 3E1M, 3IS8, 3FVB) suggesting that it serves to regulate heme potential. Replacing it with an aliphatic residue is expected to eliminate this negative effect. Another potential modification is replacement of Asp50. In native BFR Asp50 forms a salt bridge with Lys53. Replacement of Asp50 with a polar or even basic aminoacid would break this salt bridge without affecting the interaction between the two monomers. This would facilitate the interaction of Lys53 with the carboxylic acids of ZnCe_6_ potentially neutralizing the effect of their negative charge.

In summary we have determined a number of protein structural factors contributing to the redox potential of ZnCe_6_ in BFR-RC, and by emulating some of the features of PSII it may be possible to raise the potential by several hundred millivolts, but not likely all the way up to the 1.2 V required to oxidize water.

## Supporting Information

Figure S1 **Schematic diagram of atom names for ZnCe_6_.**
(TIF)

Figure S2 **Experimental vs. calculated E_m_'s of Zn-chlorin derivatives.** DFT systematically underestimates values by 0.1 V, but after correction for this systematic shift all computed values are within 20 mV from measured. Experimental E_m_'s of the following Zn-chlorins were used: ZnC, ZnC -A^3^M^10^A^13^, ZnC -M^10^, ZnC -T^5^, ZnC -P^15^, ZnC -OP, ZnC -T^5^M^10^P^15^, ZnC -P^10^, ZnC -P^3^M^10^ from [1] and ZnCe_6_ from the current work. [1] H.L. Kee, C. Kirmaier, G. Tang, J.R. Diers, C. Muthiah, M. Taniguch, J.K. Laha, M. Ptaszek, J.S. Lindsey, D.F. Bocian, D. Hoten, Effects of substituents on synthetic analogs of chlorophylls. Part 2: Redox properties, optical spectra and electronic structure, Photochem. Photobiol. 83 (2007) pp. 1125–1143.
(TIF)

Table S1 **Atomic charges for neutral and oxidized ZnCe_6_.** The charges were derived using RESP-A1 procedure (HF/6-31G*, Connolly surface, 2 RESP stages, qwt = 0.0005/0.001). Cavity radii for Poisson-Boltzmann electro-statics computations: Zn = 1.47, C = 1.7, O = 1.4, N = 1.5, H = 1.0. PARSE charges for –COOH groups were used for computation of pKas.
(DOCX)

Model S1 **The QM/MM optimized structure of the BFR-RC with bound ZnCe_6_.**
(PDB)
