# Supplementary figures and images for: Factors Controlling the Redox Potential of ZnCe6 in an Engineered Bacterioferritin Photochemical ‘Reaction Centre’
Source: PLoS One. 2013 Jul 30;8(7):e68421. doi: 10.1371/journal.pone.0068421 (PMC3728335; doi:10.1371/journal.pone.0068421)

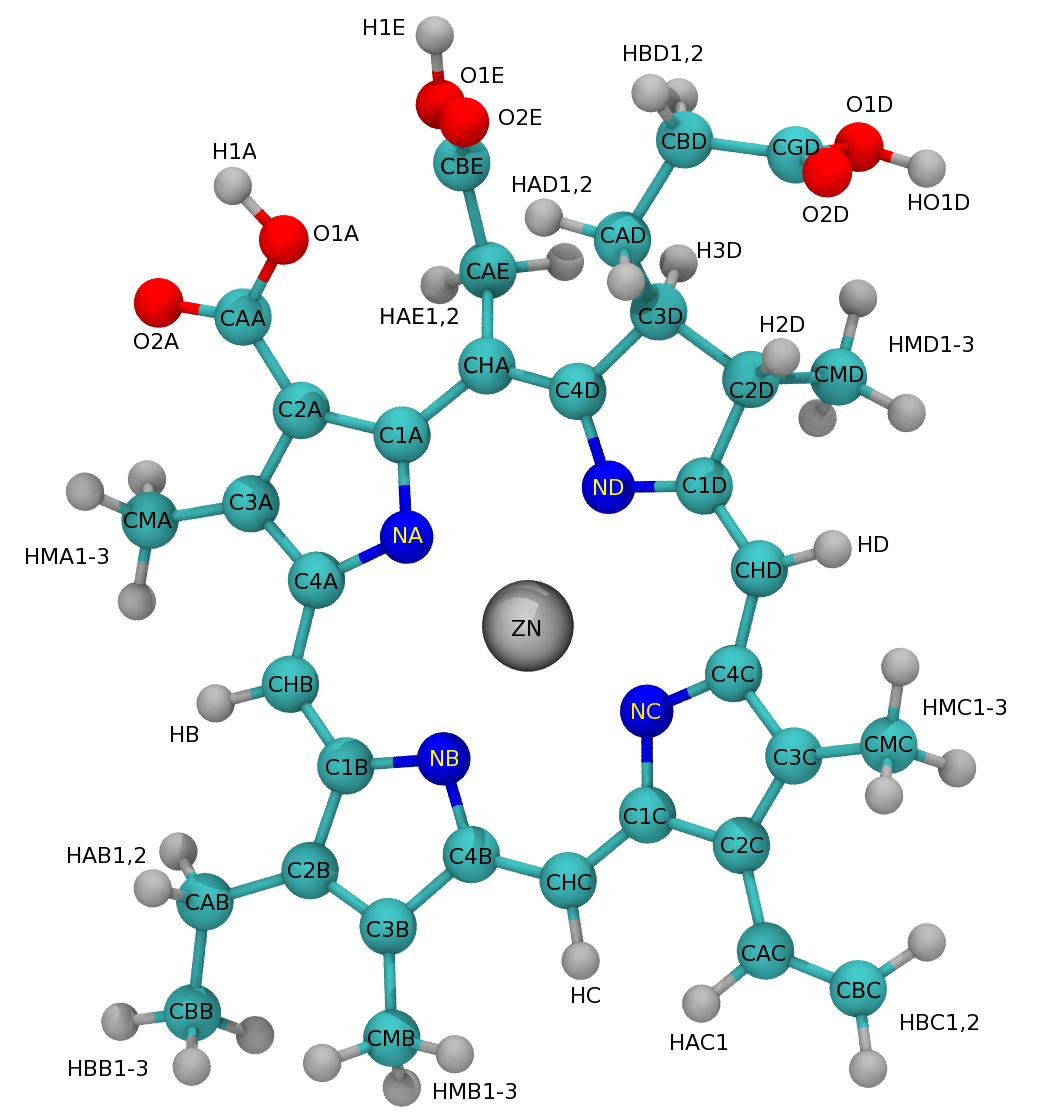

Supplement: Figure S1 — Schematic diagram of atom names for ZnCe6. (TIF) [file pone.0068421.s001.tif]
