# Supplementary material for: Factors Controlling the Redox Potential of ZnCe6 in an Engineered Bacterioferritin Photochemical ‘Reaction Centre’
Source: PLoS One. 2013 Jul 30;8(7):e68421. doi: 10.1371/journal.pone.0068421 (PMC3728335; doi:10.1371/journal.pone.0068421)
Supplement: Table S1 — Atomic charges for neutral and oxidized ZnCe6. The charges were derived using RESP-A1 procedure (HF/6-31G*, Connolly surface, 2 RESP stages, qwt = 0.0005/0.001). Cavity radii for Poisson-Boltzmann electro-statics computations: Zn = 1.47, C = 1.7, O = 1.4, N = 1.5, H = 1.0. PARSE charges for –COOH groups were used for computation of pKas. (DOCX) [file pone.0068421.s003.docx]

Table S1. Atomic charges for neutral and oxidized ZnCe_6_. The charges were derived using RESP-A1 procedure (HF/6-31G*, Connolly surface, 2 RESP stages, qwt = 0.0005/0.001). Cavity radii for Poisson-Boltzmann electro-statics computations: Zn=1.47, C=1.7, O=1.4, N=1.5, H=1.0. PARSE charges for –COOH groups were used for computation of pKas.

| # | Atom  name | Charge  reduced | Charge  oxidized |
| --- | --- | --- | --- |
| **1**  **2**  **3**  **4**  **5**  **6**  **7**  **8**  **9**  **10**  **11**  **12**  **13**  **14**  **15**  **16**  **17**  **18**  **19**  **20**  **21**  **22**  **23**  **24**  **25**  **26**  **27**  **28**  **29**  **30** | **ZN**  **NA**  **C1A**  **C2A**  **C3A**  **C4A**  **CHA**  **NB**  **C1B**  **C2B**  **C3B**  **C4B**  **CHB**  **HB**  **NC**  **C1C**  **C2C**  **C3C**  **C4C**  **CHC**  **HC**  **ND**  **C1D**  **C2D**  **H2D**  **C3D**  **H3D**  **C4D**  **CHD**  **HD** | **1.153119**  **-0.500472**  **0.029232**  **-0.078838**  **0.043641**  **0.037627**  **-0.004483**  **-0.446619**  **-0.031386**  **-0.057905**  **0.067260**  **0.083702**  **-0.020760**  **0.131281**  **-0.545711**  **0.063483**  **0.041989**  **0.082014**  **0.245178**  **-0.106474**  **0.106884**  **-0.476973**  **0.191954**  **0.111139**  **0.042283**  **0.018045**  **0.078805**  **0.040879**  **-0.382213**  **0.139720** | **1.135671**  **-0.523643**  **0.146484**  **-0.025960**  **0.056489**  **0.195239**  **-0.022431**  **-0.442603**  **0.059164**  **-0.081145**  **0.106337**  **0.212731**  **-0.025440**  **0.093844**  **-0.588825**  **0.107711**  **0.050908**  **0.098751**  **0.287763**  **-0.114445**  **0.106817**  **-0.440841**  **0.166771**  **0.110710**  **0.063478**  **0.019635**  **0.104812**  **0.049045**  **-0.358231**  **0.147613** |
| **31**  **32**  **33**  **34** | **CMA**  **HMA1**  **HMA2**  **HMA3** | **-0.259038**  **0.093060**  **0.093060**  **0.093060** | **-0.378873**  **0.144193**  **0.144193**  **0.144193** |
| **35**  **36**  **37**  **38** | **CMB**  **HMB1**  **HMB2**  **HMB3** | **-0.232209**  **0.075913**  **0.075913**  **0.075913** | **-0.268436**  **0.104880**  **0.104880**  **0.104880** |

| # | Atom  name | Charge  reduced | Charge  oxidized |
| --- | --- | --- | --- |
| **39**  **40**  **41**  **42** | **CMC**  **HMC1**  **HMC2**  **HMC3** | **-0.208352**  **0.071852**  **0.071852**  **0.071852** | **-0.208295**  **0.082916**  **0.082916**  **0.082916** |
| **43**  **44**  **45**  **46** | **CMD**  **HMD1**  **HMD2**  **HMD3** | **-0.207389**  **0.061232**  **0.061232**  **0.061232** | **-0.215648**  **0.073863**  **0.073863**  **0.073863** |
| **47**  **48**  **49**  **50**  **51**  **52**  **53** | **CAB**  **HAB1**  **HAB2**  **CBB**  **HBB1**  **HBB2**  **HBB3** | **-0.017080**  **0.033666**  **0.033666**  **-0.022956**  **0.013844**  **0.013844**  **0.013844** | **-0.020219**  **0.049637**  **0.049637**  **-0.022505**  **0.022816**  **0.022816**  **0.022816** |
| **54**  **55**  **56**  **57**  **58** | **CAC**  **HAC1**  **CBC**  **HBC1**  **HBC2** | **-0.169178**  **0.150771**  **-0.321297**  **0.156711**  **0.169478** | **-0.193477**  **0.155417**  **-0.281741**  **0.165305**  **0.164342** |
| **59**  **60**  **61**  **62** | **CAA**  **O1A**  **O2A**  **H2A** | **0.653594**  **-0.556759**  **-0.556759**  **0.430379** | **0.737304**  **-0.556759**  **-0.556759**  **0.430379** |
| **63**  **64**  **65**  **66**  **67**  **68**  **69** | **CAE**  **HAE1**  **HAE2**  **CBE**  **O1E**  **O2E**  **H2E** | **-0.029570**  **0.056834**  **0.056834**  **0.544857**  **-0.556759**  **-0.556759**  **0.430379** | **-0.132547**  **0.086955**  **0.086955**  **0.608873**  **-0.556759**  **-0.556759**  **0.430379** |
| **70**  **71**  **72**  **73**  **74**  **75**  **76**  **77**  **78**  **79** | **CAD**  **HAD1**  **HAD2**  **CBD**  **HBD1**  **HBD2**  **CGD**  **O1D**  **O2D**  **HO1D** | **-0.075040**  **0.042558**  **0.042558**  **-0.046107**  **0.033979**  **0.033979**  **0.624042**  **-0.556759**  **-0.556759**  **0.430379** | **-0.145945**  **0.067811**  **0.067811**  **-0.066981**  **0.053151**  **0.053151**  **0.655322**  **-0.556759**  **-0.556759**  **0.430379** |
